# Interosseous-lumbrical adhesions secondary to an infection: a case report

**DOI:** 10.1186/1752-1947-8-301

**Published:** 2014-09-09

**Authors:** Daniel Muder, Torbjörn Vedung

**Affiliations:** 1 Department of Hand Surgery, Uppsala University Hospital, Uppsala SE-751 85, Sweden; 2 Department of Surgical Sciences, Uppsala University, Uppsala SE-751 85, Sweden

**Keywords:** Adhesions, Chronic pain, Hand, Infection, Intermetacarpal space, Saddle deformity, Saddle syndrome

## Abstract

**Introduction:**

Adhesions between the tendons to the interosseous muscles, the lumbrical muscles and occasionally the deep transverse metacarpal ligament can be symptomatic and cause chronic discomfort in the distal part of the hand. Reports about the condition are rare and the causal factors in previous publications are in principle limited to crush injuries and contusion from a direct blow to the hand. We present a case with typical clinic findings secondary to an infection after a cat bite. To the best of our knowledge symptomatic interosseous-lumbrical adhesions caused by an infection has never been described previously.

**Case presentation:**

Our case report describes a 25-year-old Caucasian woman with chronic pain and swelling between her second and third metacarpal heads. Symptoms occurred especially under stress and developed secondary to an infection after a cat bite. Surgical exploration revealed localized adhesions between her second lumbrical muscle, her first palmar interosseous muscle and her deep transverse metacarpal ligament. The symptoms were completely relieved by surgical release of the adhesions, partial resection of the deep transverse metacarpal ligament and immediate postoperative physiotherapy.

**Conclusions:**

Physicians involved in hand surgery should be aware of the condition and look for it in patients complaining about distal intermetacarpal pain. The major causal factors for developing symptomatic interosseous-lumbrical adhesions are crush injuries or contusion to the distal part of the hand but it may also occur after an infection.

## Introduction

Watson *et al*. described posttraumatic interosseous-lumbrical adhesions and titled the condition “saddle deformity” [1,2]. The topographical anatomy of the lumbrical muscles, interosseous muscles and the deep transverse metacarpal ligament (dTML) explains this name. The lumbrical muscles on the volar side and the interosseous muscles on the dorsal side of the dTML converge just distal to the ligament and are in immediate connection to each other as they insert into the extensor hood mechanism. This lumbrical-interosseous junction slides proximally towards the dTML during intrinsic muscle contraction. The two muscle bellies can be pictured as two legs on each side of the dTML (the saddle) [2]. Adhesions can occur between two separate structures or all structures in the web-space at the level of the metacarpal head [3].

The causal factors in previous descriptions of symptomatic interosseous-lumbrical adhesion are in principle limited to contusion and crush injuries to the distal part of the hand [1-3].

We present a case with typical clinical findings secondary to an infection after a cat bite.

## Case presentation

**Figure 1:**
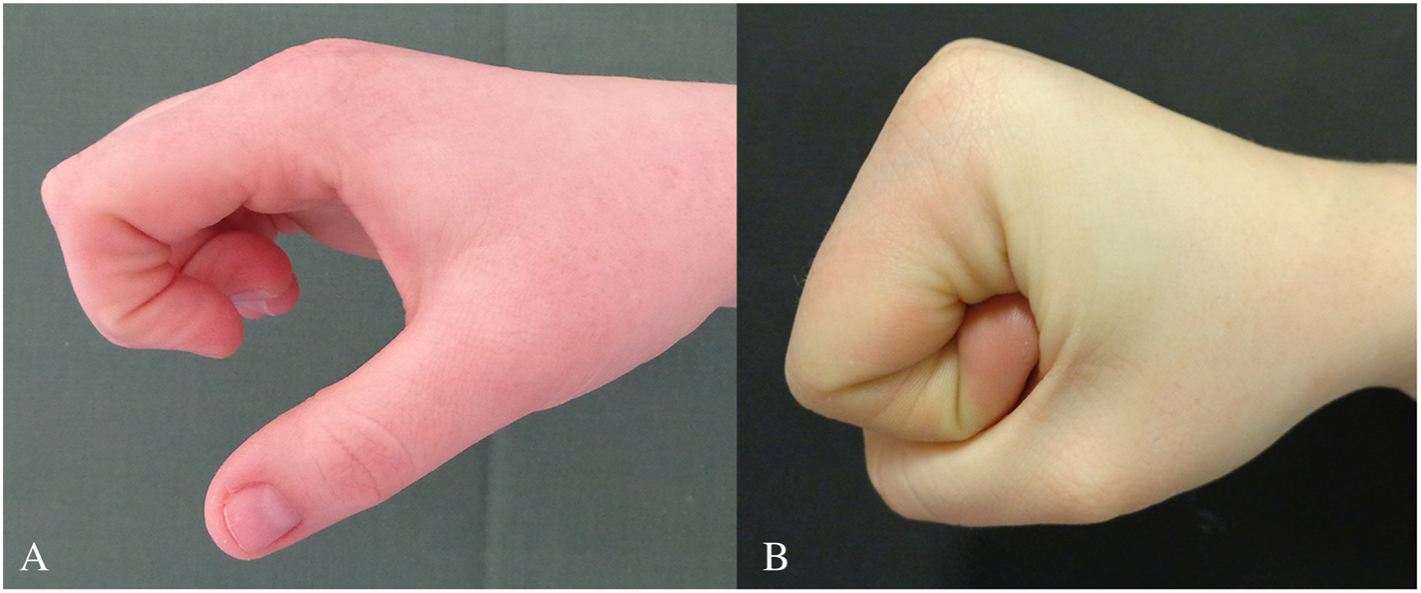
Flexion ability in the fingers, before (A) and after (B) surgical release of the interosseous-lumbrical adhesions.

A 25-year-old Caucasian woman was bitten by a cat dorsal to the metacarpophalangeal (MCP) joint to her right index finger. She was admitted to our clinic with signs of local infection and a possible septic arthritis. Her wound was debrided and irrigated and a partial rupture of her extensor indicis proprius tendon was sutured. The MCP II joint was not affected. With antibiotics and two additional irrigations her wound healed.Over time, chronic discomfort and recurrent episodes of pain and swelling developed in the area. When she was readmitted to our clinic 5 years later she was unable to work (pet-hospital) and her chief complaint was volar and dorsal pain in the distal intermetacarpal space between her second and third finger rays. Conservative treatment such as anti-inflammatory drugs, physiotherapy or immobilization had been insufficient. The range of motion (ROM) in her MCP II-III joints was painful and limited to 0/40 (extension/flexion; Figure 1A). Ultrasonography and magnetic resonance imaging (MRI) were normal. Interosseous-lumbrical adhesions involving the dTML were suspected as the symptoms increased in the intrinsic minus and the intrinsic plus finger positioning.

Under general anaesthesia, the volar aspect of the painful area was exposed by a zigzag incision as described by Watson *et al*. [1]. Adhesions were found between the second lumbrical muscle, the first palmar interosseous muscle, and the dTML. Passive intraoperative motion of her fingers demonstrated impaired excursion and inability of her muscles to move free in relation to the dTML. Release of the adhesions and resection of the distal third of the dTML resulted in normal passive excursion of her muscles without impinging the ligament. Immediate postoperative physiotherapy followed. Six weeks postoperative her hand was pain free and had full ROM (Figure 1B). The result was consistent over time with normal hand function at the final 12-month follow-up.

## Discussion

Adhesions between the intrinsic muscles distal to, or at the level of the dTML have been described in detail previously [1,2]. In previous reports, the condition is almost exclusively secondary to contusion and crush injuries to the distal part of the hand [1-3]. Of the cases in the comprehensive Chicarilli report, 10% are referred to as miscellaneous [2]. Other exceptions are local inflammation [4] and inflammation after a complex regional pain syndrome [5]. The symptomatic interosseous-lumbrical adhesion in our case was caused by a local infection.

Two different kinds of adhesions have been described [2]. The first category consists of adhesions between the lumbrical muscle and the interosseous muscles distal to and without involvement of the dTML. The adhesion influences the proximal excursion of the lumbrical-interosseous junction as it impinges the dTML during flexion in the MCP joint and extension in the interphalangeal joints; that is, the intrinsic plus finger positioning. In the second category the intrinsic muscles are stuck to the dTML or the MCP joint capsule, causing pain during flexion in the interphalangeal joints and extension in the MCP joint; that is, the intrinsic minus finger positioning (Bunnell’s test). In our case the symptoms increased in the intrinsic minus and the intrinsic plus finger positioning indicating interosseous-lumbrical adhesions involving the dTML, which also was confirmed at surgery.

Topper reported a variant of the second type of adhesion in 1997 [4]. In this single case report the adhesion was located on the radial side of the index finger involving the MCP joint capsule, the first lumbrical muscle and the first dorsal interosseous muscle. There is no dTML (“saddle”) at this site. Thus, the term “saddle deformity” can be misleading, and since there is no actual deformity the term is probably best avoided in general.

The average time between injury and surgical release in the Chicarilli series was 19 months (range 3 to 120 months), which indicates difficulties in making the diagnosis [2]. The sparse reports in the literature may indicate that it is an uncommon condition. However, as indicated by the high figures in the Chicarilli report and as mentioned in the original report, the condition is probably relatively common but not recognized [1,2]. It is important to remember and look for the condition. The relative shortage of objective findings can prohibit making the diagnosis [2].

The area where the adhesions occur may be very limited making visualization with MRI difficult [4]. With high quality MRI Tan *et al*. were able to present two cases with positive findings that correlated with the clinical diagnosis and intraoperative findings [3]. Although the findings may be subtle, they recommended an MRI in all patients suspected of having the diagnosis [3]. Also van der Veen *et al*. reported a case with MRI findings suggesting the diagnosis [5]. The MRI in our case was not helpful, other than excluding differential diagnosis. We think that the diagnosis is primarily made by clinical examination. Surgical release of the adherences is a simple procedure with a high success rate. Chicarilli *et al*. reported significant improvement in 87% of the patients (58% excellent and 29% good results) [2]. The hand function in our case was completely normalized after surgery.

## Conclusions

Physicians involved in hand surgery should be aware of the condition and look for it in patients complaining about distal intermetacarpal pain. The major casual factors for developing symptomatic interosseous-lumbrical adhesions are crush injuries or contusion to the hand but it may also occur after an infection.

## Consent

Written informed consent was obtained from the patient for publication of this case report and accompanying images. A copy of the written consent is available for review by the Editor-in-Chief of this journal.

## Abbreviations

dTML: Deep transverse metacarpal ligament; MCP: Metacarpophalangeal joint; MRI: Magnetic resonance imaging; ROM: Range of motion.

## Competing interests

The authors of this article have received grants from Uppsala county council in order to support their research. No other support was received and the authors have no conflict of interests.

## Authors’ contributions

Both authors contributed equally to the work presented in this case report. Both authors read and approved the final manuscript.
